# Nasal glomangiomyoma: a case report and review of superficial facial glomus tumors

**DOI:** 10.3389/fmed.2026.1960678

**Published:** 2026-10-19

**Authors:** Austėja Kindurytė-Šepetienė, Patricija Glovackaitė, Dominykas Markevičius, Evelina Šidlovska, Giedrė Stundžaitė-Baršauskienė, Nerijus Jakutis

**Affiliations:** 1 Faculty of Medicine, Institute of Clinical Medicine, Clinic of Rheumatology, Orthopedics, Traumatology and Reconstructive Surgery, Vilnius University, Vilnius, Lithuania; 2 Faculty of Medicine, Vilnius University, Vilnius, Lithuania; 3 Vilnius University Hospital Santaros Klinikos, National Center of Pathology, Vilnius, Lithuania

**Keywords:** case report, extradigital glomus tumor, facial soft tissue tumor, glomangiomyoma, glomus tumor, nasal tumor

## Abstract

Glomus tumors are uncommon pericytic neoplasms that most frequently occur in the subungual regions of the extremities, while superficial facial localization is rare and may present a diagnostic challenge. We report a nasal glomangiomyoma in a 63-year-old woman presenting as a painless, slowly enlarging subcutaneous mass of the upper right nasal sidewall that was clinically suspected to represent a lipoma. The lesion was completely excised, with the closest resection margin free of tumor at a microscopic clearance of 0.5 mm. Histopathological examination demonstrated vascular spaces surrounded by uniform epithelioid glomus cells together with fascicles of elongated spindle cells showing smooth-muscle differentiation and a gradual morphological transition between the two components. Immunohistochemically, the glomus cells expressed smooth muscle actin, while the smooth-muscle component showed strong desmin positivity; proliferative activity was low. These findings supported the diagnosis of glomangiomyoma. No clinical evidence of recurrence was observed at 6-month follow-up. A focused review of the literature identified a limited number of reported superficial facial glomus tumors, many of which presented without the characteristic pain associated with digital lesions. This case highlights that glomus tumors should be considered in the differential diagnosis of persistent superficial facial nodules, including clinically benign-appearing lesions, and emphasizes the importance of histopathological evaluation for definitive diagnosis and appropriate classification.

## 1 Introduction

Glomus tumors are uncommon benign mesenchymal neoplasms arising from modified smooth muscle cells of the glomus body, a specialized arteriovenous structure involved in thermoregulation (1). They account for approximately 1.6% of soft tissue tumors and most commonly occur in the subungual regions of the extremities (2). Extradigital glomus tumors are considerably less frequent and may present a diagnostic challenge because of their atypical location and nonspecific clinical features (3).

According to the 2020 World Health Organization (WHO) classification of soft tissue tumors, glomus tumors are classified within the pericytic (perivascular) tumor group, which also includes myopericytoma and angioleiomyoma, among other entities (4). Within the glomus tumor category, WHO recognizes glomangioma, glomangiomyoma, glomangiomatosis, glomus tumor of uncertain malignant potential, and malignant glomus tumor (4). Glomangiomyoma is characterized by morphological evidence of smooth-muscle differentiation, with transition from conventional rounded glomus cells to elongated spindle-shaped cells exhibiting mature smooth-muscle features (5). Recognition of this morphology is important because smooth muscle actin expression is characteristic of glomus tumors more broadly and, by itself, does not establish the diagnosis of glomangiomyoma.

Glomus tumors involving the facial region are exceptionally uncommon (3). Reported superficial facial lesions have involved the cheek, lip, eyelid, forehead, and nasal soft tissues. In contrast to classical subungual glomus tumors, which characteristically present with severe pain, pinpoint tenderness, and cold sensitivity, facial lesions may be painless and present only as slowly enlarging superficial nodules (6). Consequently, they may clinically resemble considerably more common benign facial soft-tissue lesions, resulting in low clinical suspicion and delayed or unexpected diagnosis.

Definitive diagnosis relies on histopathological examination, supported by immunohistochemistry. Microscopically, glomus tumors typically consist of uniform rounded glomus cells arranged around vascular channels (7). Immunohistochemically, tumor cells generally express smooth muscle actin (SMA), consistent with their perivascular myoid differentiation (8). Complete surgical excision is the treatment of choice, with recurrence reported predominantly in association with incomplete excision (9).

We present a case of glomangiomyoma arising in the superficial nasal soft tissues of a 63-year-old woman, clinically suspected to represent a lipoma and diagnosed following surgical excision and histopathological evaluation. We additionally performed a focused review of published superficial facial glomus tumors to characterize their clinical presentation, anatomical distribution, histopathological features, management, and reported outcomes. By highlighting the potential for painless facial glomus tumors to mimic common benign soft-tissue lesions, this report emphasizes their consideration in the differential diagnosis of persistent superficial facial nodules.

## 2 Case description

A 63-year-old woman presented with a slowly enlarging subcutaneous lesion located in the upper right nasal sidewall. The lesion had first been noticed approximately 3 years earlier and had gradually increased in size. It was painless and was not associated with tenderness, cold sensitivity, bleeding, skin discoloration, or other local symptoms.

Clinical examination revealed a small, well-circumscribed subcutaneous mass in the right nasal region without overlying skin changes or signs of inflammation. Based on its clinical appearance, the lesion was initially suspected to represent a lipoma. Given its small size and clinically benign appearance, no preoperative imaging was performed. Surgical excision under local anesthesia was undertaken for diagnostic and therapeutic purposes.

Macroscopically, the excised specimen consisted of a grayish soft-tissue fragment measuring 0.7 × 0.5 × 0.4 cm. Histopathological examination demonstrated a well-circumscribed subcutaneous neoplasm composed of variably sized vascular spaces surrounded by uniform rounded epithelioid glomus cells with small round nuclei and eosinophilic cytoplasm. Intermixed with the epithelioid glomus cells were fascicles of spindle-shaped smooth-muscle cells with abundant eosinophilic cytoplasm and elongated, cigar-shaped nuclei. A gradual morphological transition from the rounded epithelioid glomus cells to the spindle-shaped cells exhibiting smooth-muscle differentiation was identified within the lesion (Figure 1A). No significant cytological atypia, tumor necrosis, or increased mitotic activity was observed.

**Figure 1:**
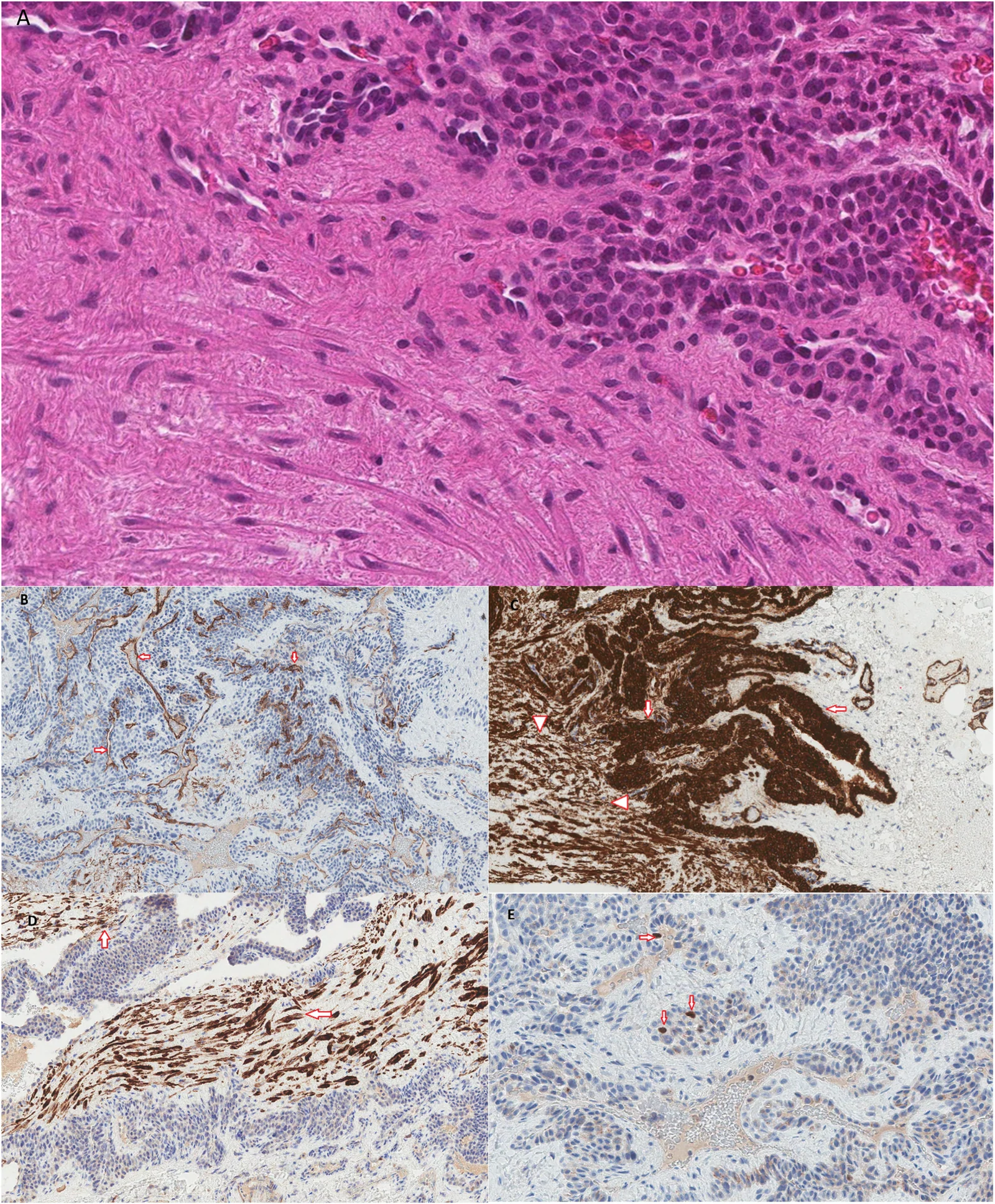
Histopathological examination and immunohistochemistry. **(A)** Hematoxylin and eosin (H&E) stained section showing transition from rounded glomus cells to elongated spindle cells with smooth-muscle differentiation (magnification, ×20). **(B)** CD34 IHC highlighted the endothelial lining of vascular channels (magnification, ×20). **(C)** Cytoplasmic positivity for smooth muscle actin (SMA) in glomus cells indicated by the arrow and smooth muscle differentiation indicated by arrowhead (magnification, ×20). **(D)** The smooth muscle component demonstrates strong positivity for Desmin, whereas the glomus cells are negative (magnification, ×20). **(E)** Ki-67 IHC: Scattered tumor cell nuclei show Ki-67 positivity, indicating low proliferative activity (magnification, ×40).

Immunohistochemically, the glomus cells demonstrated diffuse cytoplasmic positivity for smooth muscle actin (SMA) (Figure 1C), while CD34 highlighted the endothelial lining of the vascular channels (Figure 1B). The spindle-shaped smooth-muscle component demonstrated strong desmin positivity, whereas the rounded glomus cells were desmin-negative (Figure 1D). The Ki-67 proliferation index was <1% (Figure 1E). The combination of conventional glomus tumor morphology with a gradual transition toward a morphologically and immunohistochemically identifiable smooth-muscle component supported the diagnosis of glomangiomyoma. The closest resection margin was free of tumor, with a microscopic clearance of 0.5 mm.

The principal clinical events from lesion onset to postoperative follow-up are summarized in Table 1.

**Table 1:** Clinical timeline of the present case.

Time point	Clinical event
Approximately 3 years before presentation	Patient first noticed a small, painless subcutaneous lesion of the upper right nasal sidewall, which gradually increased in size.
Clinical presentation	Small, well-circumscribed, painless subcutaneous mass without overlying skin changes, tenderness, cold sensitivity, or bleeding; clinically suspected to represent a lipoma.
Surgical treatment	No preoperative imaging was performed because of the lesion's small size and clinically benign appearance. The lesion was excised under local anesthesia for diagnostic and therapeutic purposes.
Histopathological assessment	Histopathological and immunohistochemical findings demonstrated glomangiomyoma. The closest resection margin was free of tumor, with a microscopic clearance of 0.5 mm.
6 months after excision	Surgical site was well healed, with no clinical evidence of local recurrence or postoperative complications.

The postoperative course was uneventful. At clinical follow-up 6 months after excision, the surgical site was well healed, with no clinical evidence of local recurrence or postoperative complications.

The case is reported in accordance with the CARE guidelines, and the completed CARE checklist is provided as Supplementary Material.

## 3 Literature review

To contextualize the present case, a focused literature review of published superficial facial glomus tumors was conducted. PubMed, Scopus, and Google Scholar were searched using combinations of the terms “glomus tumor,” “glomangioma,” “glomangiomyoma,” “facial,” “face,” “cheek,” “lip,” “nasal,” “nose,” “forehead,” “chin,” “eyelid,” and “periorbital.” The search was last updated in September 2026. Reference lists of eligible articles were also manually screened to identify additional reports.

Original case reports and case series describing superficial glomus tumors involving the facial soft tissues, including the cheek, lips, nasal soft tissues, forehead, chin, eyelid, and periorbital region, were considered eligible. Articles published in English, French, or Lithuanian were included when sufficient case-level clinical and pathological information could be retrieved from the full text or, when the full text was unavailable, from an accessible abstract. Lesions arising within the nasal cavity or sinonasal tract, including the nasal septum, were excluded because the review was restricted to superficial facial soft-tissue tumors. Reports lacking sufficient information to confirm the anatomical location or diagnosis were also excluded.

Eligible reports were reviewed for the number of patients, age, sex, anatomical location, clinical presentation, symptom duration, lesion size, histopathological diagnosis, and reported follow-up and recurrence. Data not provided in the original publication were recorded as not reported (NR) rather than inferred. The identified cases and the present case are summarized descriptively in Table 2. Given the rarity of these tumors and the predominantly case-based literature, this review was intended as a focused descriptive review rather than a systematic review or quantitative synthesis.

**Table 2:** Clinical and pathological characteristics of published cases of superficial facial glomus tumors identified in the literature review.

Study	Number of patients	Age, years	Sex	Symptoms	Duration of symptoms	Location	Size (cm)	Histopathological diagnosis	Recurrence (follow-up time)
Maurya et al., 2025 (10)	1	50	F	Pain on accidental touch	18 months	2 cm inferior to the medial canthus of the right eye.	0.4	Glomus tumor	No (3 months)
Lanza et al., 2005 (11)^a^	1	65	M	N.R.	N.R.	Lower lip	N.R.	N.R.	N.R.
Charles, 1976 (12)^a^	1	17	N.R.	Painless/nontender multiple lesions	N.R.	Face, eyelid, anterior orbit, and palate	N.R.	Glomus tumors	N.R.
Veros et al., 2012 (13)	1	24	M	Pain and local tenderness	4 years	Left cheek	1.38 × 0.9	Glomus tumor	Yes (2 months)
Mounayer et al., 2001 (14)	7	3–26	4 M/3 F	Asymptomatic for all patients	N.R.	Cheek, lips, lower eyelid	N.R.	Glomangiomas	N.R.
Yang et al., 2002 (15)	1	25	M	Asymptomatic	Several years	Right cheek	1.5 × 1.5	Glomangiomyoma	No (23 months)
Wang et al., 2008 (16)	2	51 and 58	M and F	Asymptomatic	N.R.	Right cheek; left cheek	N.R.	Glomus tumors	N.R.
Moody et al., 1986 (17)	1	65	F	Asymptomatic swelling	Several years	Upper lip	N.R.	N.R.	N.R.
Sakashita et al., 1997 (18)	1	54	M	Painless swelling	5 years	Upper lip	N.R.	N.R.	N.R.
Yu et al., 2000 (19)	1	54	F	Asymptomatic	30 years	left mandibular area/lower lip	N.R.	N.R.	N.R.
Zhang and Zhou, 2026 (20)	1	28	M	Asymptomatic	1 year	Left cheek	0.6 × 0.4 × 0.4	Benign intravascular glomus tumor	No (follow-up NR)
Present case 2026	1	63	F	Asymptomatic	3 years	Upper right nasal sidewall	0.7 × 0.5 × 0.4	Glomangiomyoma	No clinical recurrence (6 months)

F, female; M, male; NR, not reported.

a Case-level information obtained from an accessible abstract because the full text was unavailable.

## 4 Discussion

Glomus tumors are uncommon pericytic neoplasms that predominantly occur in the distal extremities, whereas superficial facial involvement is rare. The cases identified in our focused review demonstrate a heterogeneous facial distribution, particularly involving the cheek and lips, with occasional lesions in the periorbital and nasal regions. Importantly, facial glomus tumors may differ clinically from their classical digital counterparts. Although digital lesions are characteristically associated with pain, localized tenderness, and cold sensitivity, several reported facial tumors were painless or asymptomatic and presented primarily as slowly enlarging superficial nodules (21).

The present case illustrates the resulting diagnostic challenge. The lesion had enlarged slowly over approximately 3 years without pain, tenderness, cold sensitivity, skin discoloration, or other local symptoms. Because it appeared as a small, well-circumscribed, clinically benign subcutaneous mass, a lipoma was initially suspected and no preoperative imaging was performed. The diagnosis of glomangiomyoma was therefore unexpected and was established following excision and histopathological examination. Similar atypical presentations have been documented in the literature. In a 20-year series of 56 extradigital glomus tumors, Schiefer et al. reported that cold hypersensitivity occurred in only 2% of patients and that the mean diagnostic delay exceeded 7 years; only one nasal and one cheek lesion were identified (3). Smith et al., in a review of 31 oral and paraoral glomus tumors, identified the lips as the most frequent site and reported pain or tenderness in fewer than half of cases (5). More recently, Zhang and Zhou described a painless superficial cheek glomus tumor that was initially misclassified on ultrasonography and diagnosed definitively only after histopathological and immunohistochemical evaluation (20). These observations emphasize that the absence of the characteristic painful presentation does not exclude a glomus tumor in the facial region.

According to the 2020 World Health Organization (WHO) classification of soft tissue tumors, glomus tumors are classified within the pericytic (perivascular) tumor group, which also includes myopericytoma and angioleiomyoma, among other entities (4). Within the glomus tumor category, WHO recognizes glomangioma, glomangiomyoma, glomangiomatosis, glomus tumor of uncertain malignant potential, and malignant glomus tumor (4). The diagnosis of glomangiomyoma in the present case was based on morphological evidence of smooth-muscle differentiation rather than SMA expression alone. In addition to uniform rounded epithelioid glomus cells surrounding vascular spaces, the tumor contained fascicles of spindle-shaped cells with abundant eosinophilic cytoplasm and elongated, cigar-shaped nuclei, with a gradual morphological transition between the epithelioid glomus and smooth-muscle components. The latter demonstrated strong desmin positivity, whereas the rounded glomus cells were desmin-negative. These findings support the smooth-muscle differentiation characteristic of glomangiomyoma. This distinction is important because SMA expression is typical of glomus tumors more broadly and is therefore insufficient by itself to establish the diagnosis of glomangiomyoma.

The histopathological differential diagnosis includes other perivascular and smooth-muscle neoplasms, particularly myopericytoma and angioleiomyoma. Conventional glomus tumors are characterized by relatively uniform rounded cells arranged around vascular channels, whereas the conspicuous transition from rounded glomus cells to spindle-shaped cells with mature smooth-muscle morphology supports glomangiomyoma in the present case. CD34 highlighted the vascular endothelium rather than the neoplastic glomus cells, while the very low Ki-67 proliferation index (<1%), together with the absence of significant cytological atypia, tumor necrosis, or increased mitotic activity, supported benign histological features. Head and neck paragangliomas, historically also referred to as “glomus tumors,” should not be confused with true soft-tissue glomus tumors, as they represent a distinct neuroendocrine neoplasm (22–24).

Complete surgical excision is the principal treatment for localized superficial glomus tumors. In the present case, the lesion was completely excised, and histopathological examination confirmed that the closest resection margin was free of tumor, with a microscopic clearance of 0.5 mm. The postoperative course was uncomplicated, and at 6-month clinical follow-up, the surgical site remained well healed with no clinical evidence of local recurrence. However, this follow-up remains relatively short and does not permit conclusions regarding long-term recurrence. The available facial literature is similarly limited predominantly to individual case reports and small case series, with heterogeneous and frequently incompletely reported follow-up. Accordingly, conclusions regarding recurrence rates or long-term prognosis in superficial facial glomus tumors should be interpreted cautiously.

The principal clinical value of this case is therefore not rarity alone, but the potential for an extradigital facial glomus tumor to mimic a common benign soft-tissue lesion. In our patient, the painless and clinically innocuous appearance led to an initial clinical suspicion of a lipoma, with the definitive diagnosis established only after excision and histopathological examination. Glomus tumors should therefore be considered in the differential diagnosis of persistent superficial facial nodules even when characteristic pain and cold sensitivity are absent. Histopathological examination remains essential for definitive diagnosis and, in uncommon subtypes such as glomangiomyoma, careful assessment of cellular morphology supported by appropriate immunohistochemistry is necessary for accurate classification.

## 5 Conclusion

Superficial facial glomus tumors should be considered in the differential diagnosis of persistent, slowly enlarging facial soft-tissue nodules, even in the absence of characteristic pain or cold sensitivity. As demonstrated by the present case, their clinically benign appearance may lead to suspicion of more common lesions, such as lipoma, making histopathological examination essential for definitive diagnosis. Complete surgical excision remains the treatment of choice. To our knowledge, this is the first reported case of superficial facial glomangiomyoma in Lithuania.

## Data Availability

The original contributions presented in the study are included in the article/Supplementary Material, further inquiries can be directed to the corresponding author.
